# Ruling out Pulmonary Embolism in Patients with High Pretest Probability

**DOI:** 10.5811/westjem.2017.10.36219

**Published:** 2018-03-08

**Authors:** Murtaza Akhter, Jeffrey Kline, Bikash Bhattarai, Mark Courtney, Christopher Kabrhel

**Affiliations:** * University of Arizona College of Medicine-Phoenix, Maricopa Integrated Health System, Department of Emergency Medicine, Phoenix, Arizona; † Indiana University School of Medicine, Department of Emergency Medicine and Department of Cellular and Integrative Physiology, Indianapolis, Indiana; ‡ University of Arizona College of Medicine-Phoenix, Maricopa Integrated Health System, Department of Medicine Administration, Phoenix, Arizona; § Northwestern University’s Feinberg School of Medicine, Department of Emergency Medicine, Chicago, Illinois; ¶ Massachusetts General Hospital, Department of Emergency Medicine, Center for Vascular Emergencies, Boston, Massachusetts.; || Harvard Medical School, Department of Emergency Medicine, Boston, Massachusetts

## Abstract

**Introduction:**

The American College of Emergency Physicians guidelines recommend more aggressive workup beyond imaging alone in patients with a high pretest probability (PTP) of pulmonary embolism (PE). However, the ability of multiple tests to safely rule out PE in high PTP patients is not known. We sought to measure the ability of negative computed tomography pulmonary angiography (CTPA) *along with* negative D-dimer to rule out PE in these high-risk patients.

**Methods:**

We analyzed data from a previous prospective observational study conducted in 12 emergency departments (ED). Wells score criteria were entered by providers before final PE testing. PE was diagnosed by imaging on the index ED visit, or within 45 days, demonstrating either PE or deep vein thrombosis (DVT), or if the patient died of PE during the 45-day, follow-up period. Testing threshold was set at 1.8%.

**Results:**

A total of 7,940 patients were enrolled and tested for PE, and 257 had high PTP (Wells >6). Sixteen of these high-risk patients had negative CTPA *and* negative D-dimer, of whom two were positive for PE (12.5% [95% confidence interval {2.2%–40.0%}]). One of these patients had a DVT on CT venogram and the other was diagnosed at follow-up.

**Conclusion:**

Our analysis suggests that in patients with high PTP of PE, neither negative CTPA by itself nor a negative CTPA *plus a* negative D-dimer are sufficient to rule out PE. More aggressive workup strategies may be required for these patients.

## INTRODUCTION

Pulmonary embolism (PE) is a deadly disease, often with rapid onset and ensuing precipitous decline.^1^ It is, therefore, imperative for physicians to be able to safely rule out PE. The complicated nature of the workup has led to numerous publications describing the diagnostic workup of potential PE.^2^–^11^ The American College of Physicians recommends CTPA as the first diagnostic test for patients who have a high pretest probability (PTP) of PE,^9^ with D-dimer testing not recommended as a stand-alone test to rule out PE in this group. This is also the guideline of other societies, including the American College of Radiology,^10^ the American Academy of Family Physicians,^12^ and websites such as UpToDate.^11^ The American College of Emergency Physicians is an exception, having a Level C recommendation to perform two negative tests to rule out PE in high PTP patients.^8^ In this study, we sought to validate this guideline by testing the ability of a negative CTPA *with* a negative D-dimer to rule out PE in high-risk patients.

## METHODS

We used data from a previous prospective, observational study conducted in 12 emergency departments (ED) in the United States from July 1, 2003, until November 30, 2006, using methodology previously described in a report validating the Pulmonary Embolism Rule-out Criteria (the PERC rule).^13^ This study was approved by the institutional review boards for the conduct of human subject research at all institutions. Of note, the original study included a New Zealand site; however, given the potential for practice variation between U.S. sites and a single NZ site, we limited our analysis to the U.S. sites. This is consistent with previously published work from this dataset.^14^,^15^

Patients were enrolled in the ED and included if they had signs or symptoms that the treating physician interpreted as sufficient to warrant testing for PE (at least one of the following: D-dimer blood test, CTPA, or ventilation-perfusion scan) and they indicated willingness to participate by process of informed consent. Patients were excluded if they were already being treated for venous thromboembolic disease (PE or deep venous thrombosis [DVT]) with therapeutic levels of anticoagulation as well as patients with CTPA, ventilation-perfusion scintillation, or duplex Doppler testing performed within the preceding seven days that was diagnostic of PE or DVT. Also excluded were patients with overt circulatory shock or respiratory failure, as well as those with social circumstances that have been highly predictive of loss to follow-up, including homelessness or imprisonment.

All clinical data, including signs, symptoms, and variables (including Wells score criteria), were entered before recording the results of final PE testing while patients were in the ED. Using the standard definitions of negative, Liatest, VIDAS, and MDA D-dimers were considered negative at concentrations of <500 ng / ml, Biopool Minutex at <250 ng/ mL, Hemosil at <244 ng / mL, and the advanced D-dimer at <1.6 lg/ mL. The outcome of interest was a diagnosis of acute PE during the index ED visit or within 45 days of the patient’s ED evaluation. We considered patients to have PE if they were evaluated for possible PE in the ED, and had radiologic confirmation of the diagnosis of either PE or DVT during the index visit or within 45 days of the index visit, or if they died of PE during the 45-day follow-up period. Confirmatory imaging included CTPA or conventional angiography showing a pulmonary arterial or deep venous filling defect interpreted as positive for PE or DVT, high-probability V/Q scan, or positive venous ultrasound consistent with DVT in the proximal or distal vasculature of the upper or lower extremities. All imaging results were based on the dictated report of board-certified attending radiologists not affiliated with (and blinded to) the study. Patients were followed for 45 days using a previously validated, published methodology that included chart review and telephone follow-up.^13^,^16^

**Population Health Research Capsule**

What do we already know about this issue?

Pulmonary embolism (PE) is a deadly disease, and in patients with high pretest probability (PTP) of PE, computed tomography pulmonary angiography (CTPA) can often miss PE.

What was the research question?

Is negative CTPA along with a negative D-dimer sufficient to rule out PE in high PTP patients?

What was the major finding of the study?

In patients with high PTP of PE, neither negative CTPA nor negative CTPA plus negative D-dimer is sufficient to rule out PE.

How does this improve population health?

In patients with high PTP for PE, more aggressive workup strategies may be required despite initial negative testing.

Testing threshold was set at 1.8% based on the Pauker and Kassirer method.^17^,^18^ Proportions are described with confidence intervals (CI) using mid-p exact calculations. We used Microsoft Excel for all calculations.

## RESULTS

**Figure 1:**
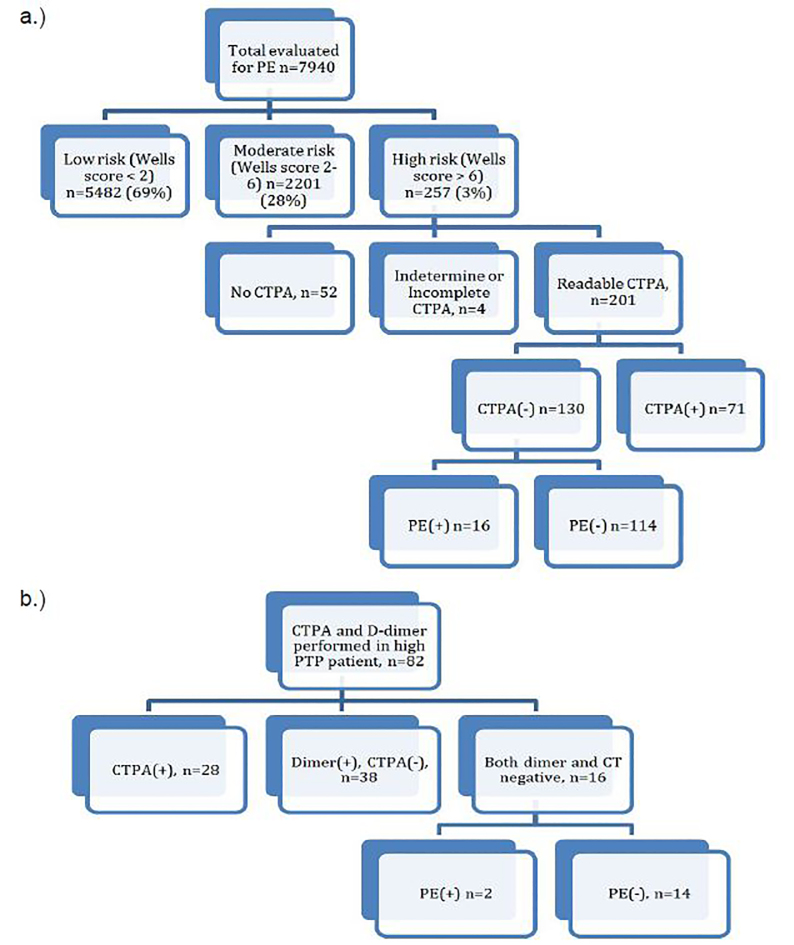
Pathway and outcomes. a)Testing and outcomes of high pretest probability patients. b) Outcomes of high pretest probability patients who had both CTPA and D-dimer *PE*, pulmonary embolism; *CTPA*, computed tomography pulmonary angiography; *PTP*, pretest probability. Note: It is simply a coincidence that the number of patients with negative CTPA who ultimately had PE (n=16) is the same as the number of patients who had both a negative CTPA and negative D-dimer (n=16).

A total of 7,940 patients were prospectively enrolled in the original study,^13^ of whom 257 had Wells score > 6 and thus had high PTP. The table shows baseline characteristics of these patients. The overall rate of PE in these high PTP patients was 37.4% (95% CI [31.5%–43.6%]). Of the 205 high PTP patients who underwent CTPA, four had CTs that were either incomplete or indeterminate. Of the remaining 201 valid CTPAs, 130 were negative for PE. Sixteen of these 130 patients, or 12.3% (95% CI [7.4%–19.5%]), were ultimately positive for PE (Figure 1a.). One of these 16 patients had an intermediate V/Q scan and a proximal clot on extremity Doppler. Seven patients had DVTs found on CT venogram. An additional two had proximal DVTs on extremity Doppler, and one had distal DVT on extremity Doppler. The remaining patients were diagnosed on follow-up.

Eighty-two of the 257 high PTP patients underwent both CTPA and D-dimer (Figure 1b). Sixteen of these patients had negative CTPA *and* negative D-dimer, and two of these 16 (12.5% [95% CI {2.2%–40.0%}]) were positive for PE. One of these patients had DVT on CT venogram, and the other was diagnosed on follow-up.

## DISCUSSION

This analysis was undertaken to determine if current guidelines can rule out PE in high PTP patients. Our analysis suggests that neither negative CTPA (by itself) nor negative CTPA *with* negative D-dimer can sufficiently rule out PE in high-risk patients. This is in line with previous research. Multiple studies have shown that CTPAs miss some PE.^19^–^22^ In the landmark PIOPED-II trial, the sensitivity of CTPA was 83%; moreover, in the subset of high-risk patients, 40% of patients with negative CTPA were diagnosed with PE or DVT.^23^ Moreover, our analysis suggests that adding a negative D-dimer to a negative CTPA may still be insufficient to rule out PE in high-risk patients.

This appears to be in contrast to literature suggesting that a D-dimer and CTPA algorithm is safe.^5^,^7^ However, studies that evaluated these algorithms included relatively small numbers of high-risk (Wells score > 6) patients, so the apparent safety of the CTPA plus D-dimer strategy may be influenced by the much larger numbers of non-high-risk patients in these studies. When stratifying for high-risk patients, all diagnostic tests have much lower abilities to rule out PE.^3^,^6^,^23^,^24^ This is supported by a recent study in which even 64-slice CTPA missed a significant number of PEs in high-risk patients,^25^ most of whom were diagnosed by additional imaging within the index visit (with the other few diagnosed during three-month clinical follow-up).^25^ Our study shows that in patients with high Wells score, not only is a negative CTPA insufficient to rule out PE, but also that a negative CTPA along with a negative D-dimer still misses a substantial number of PEs.

It is possible that newer CTs with more slices are more sensitive at picking up PEs, and therefore would yield fewer false-negative CTs. However, a Bayesian calculation using meta-analysis data of prevalence of PE in high-probability patients^26^ and CTPA sensitivity and specificity^19^ also yields an unacceptably high miss rate of 10.4% (95% credible region 6.0%– 15.3%) – similar to our empirical findings of a miss rate of 12.3% (95% CI [7.4%–19.5%]). In other words, to go from a PTP of 37.4% (this prevalence of PE in our cohort was lower than in Ceriani’s^26^ meta-analysis) to a post-test probability of 1.8%, the negative likelihood ratio (LR[−]) of the test would have to be lower than 0.03. However, a CT sensitivity of 88.9% and specificity of 94.6% (as per the meta-analysis by Hogg et al^19^) yields a LR(−) of only 0.12; other meta-analyses would yield even higher LR(−)s, and therefore make PEs even harder to rule out.^20^–^22^

Furthermore, a recent study by Moores et al.^25^ looked prospectively at outcomes in high-risk patients who underwent 64-slice CTPA. The study found that among patients with high Wells score and negative CTPA, 5.2% had PE or DVT. Therefore, even the newest CT scanners miss an unacceptable amount of PEs in high PTP patients.

It may be that some of these “missed PEs” are subsegmental PEs (SSPE). There is debate as to whether SSPEs need to be treated. On one hand, many SSPEs may not be PEs at all but radiological artifacts,^27^ and their clinical significance may be limited.^28^ On the other hand, patients with SSPE appear to have similar recurrence rates to those with proximal PEs, and have significantly higher mortality than those without PE.^29^ A finding of SSPE may require calculations of risks and benefits regarding anticoagulation, especially in those at increased risk of bleeding.^30^,^31^

The “test threshold” is meant to balance the benefits of testing (e.g., diagnoses made and treated) with the risks of testing (e.g., for CT, radiation exposure, contrast nephropathy, allergic reactions, false positive results) and to identify patients below which testing is more likely to cause harm than benefit.^17^,^18^ We used a threshold of 1.8%, which is the same threshold calculated by Kline et al.,^18^ and similar to the test threshold published by Lessler et al. (1.4%).^32^ These thresholds are also similar to the “acceptable” miss rate of pulmonary imaging, determined by the false negative rate of catheter pulmonary angiography. We acknowledge that individual physicians and patients may have their own clinical thresholds for the percentage of PE that are acceptable to miss, and we also acknowledge that the test threshold may vary over time as technology changes and risks of testing (and PE) are recalculated. However, we believe that 1.8% is a reasonable threshold that, at the least, should be reached with diagnostic testing.

## LIMITATIONS

The results of this study must be interpreted within the context of its design. Our analysis comes from data from a large, multicenter study, performed in academic and community centers, resulting in a heterogeneous population. The study was observational and noninterventional, such that we believe the results represent the real world, but probably should not be compared or contrasted to studies that purport to follow a rigid study protocol. The diagnostic criterion standard for this study was PE (or DVT) within 45 days of the index visit that was detected by standard care processes. While it is possible that a PE or DVT found during follow-up is truly a *new* thromboembolic event and that negative workup in the ED truly was negative at the time, it is standard in the literature to use diagnosis of PE or DVT during follow-up as the gold standard diagnostic criterion for negative workup in the ED. 3,5,7,19,23,25,29,33–40 The original study did not have the resources to perform radiologic testing to monitor asymptomatic patients for PE or DVT. It remains possible that a few patients had a PE or DVT and went undiagnosed during the follow-up period, and these patients were incorrectly classified as true-negatives.

Since this was a multicenter trial, multiple different D-dimers were used. We feel this strengthens the generalizability of our findings. However, although our data analysis did not suggest this, it is possible that some assays are more prone to false negatives than others.

Despite the fact a large number of patients were enrolled, relatively few patients had a high Wells score. This is consistent with observations from our prior work.^15^,^33^ The relatively small number of patients with Wells score > 6 may be why our empiric data revealed only 16 high-risk patients with negative CTPA *and* negative D-dimer. However, while this particular sample size led to a wide confidence interval, this 95% CI still did not cross the 1.8% threshold at which further workup for PE can be stopped.^18^,^32^,^41^

## CONCLUSION

Our study suggests that in patients with high pre-test probability for PE, a negative CT should be interpreted with caution, and that even *two* high-sensitivity tests may be insufficient to rule out PE in these high-risk patients. Further studies should evaluate long-term outcomes in high PTP patients – in particular, those who have been “ruled out” by diagnostic testing.

## Figures and Tables

**Table:** Characteristics of patients enrolled in 12 emergency departments across the United States presenting with signs or symptoms suggestive of high risk (Wells score > 6) of pulmonary embolism (n=257).

Demographics	% or Mean	95% Confidence Interval	
Age	52.8 [range 17–91]	50.6	54.9
Female	54.9% (141/257)	48.7%	60.9%
White	61.1% (157/257)	55.0%	66.9%
Black	30.4% (78/257)	25.0%	36.2%
Hispanic	6.2% (16/257)	3.7%	9.7%
Asian	0.8% (2/257)	0.1%	2.5%
Other race	1.6% (4/257)	0.5%	3.7%
